# Normal tissue studies in radiation oncology: A systematic review of highly cited articles and citation patterns

**DOI:** 10.3892/ol.2014.2252

**Published:** 2014-06-13

**Authors:** CARSTEN NIEDER, NICOLAUS H. ANDRATSCHKE, ANCA L. GROSU

**Affiliations:** 1 Department of Oncology and Palliative Medicine, Nordland Hospital, Bodø 8092, Norway; 2 Institute of Clinical Medicine, Faculty of Health Sciences, University of Tromsø, Tromsø 9038, Norway; 3 Department of Radiation Oncology, University Hospital Rostock, Rostock D-18059, Germany; 4 Department of Radiation Oncology, University Hospital Freiburg, Freiburg D-79106, Germany

**Keywords:** radiotherapy, radiation oncology, normal tissue, side effects, citation, research evaluation

## Abstract

Radiation therapy is one of the cornerstones of modern multidisciplinary cancer treatment. Normal tissue tolerance is critical as radiation-induced side effects may compromise organ function and quality of life. The importance of normal tissue research is reflected by the large number of scientific articles, which have been published between 2006 and 2010. The present study identified important areas of research as well as seminal publications. The article citation rate is among the potential indicators of scientific impact. Highly cited articles, arbitrarily defined as those with ≥15 citations, were identified via a systematic search of the citation database, Scopus. Up to 608 articles per year were published between 2006 and 2010, however, <10% of publications in each year accumulated ≥15 citations. This figure is notably low, when compared with other oncology studies. A large variety of preclinical and clinical topics, including toxicity prediction, the dose-volume relationship and radioprotectors, accumulated ≥15 citations. However, clinical prevention or mitigation studies were underrepresented. The following conclusion may be drawn from the present study; despite the improved technology that has resulted in superior dose distribution, clinical prevention or mitigation studies are critical and must receive higher priority, funding and attention.

## Introduction

Due to the infiltrative nature of malignant tumors and the resulting requirement for safety margins surrounding macroscopic lesions, as well as tumor motion and set-up variations, radiation treatment inevitably influences surrounding normal tissues. Due to the potentially serious consequences of normal tissue damage, significant investigations have been directed towards improving the therapeutic index (1–4). As a result of feedback from previous studies, clinical studies regarding normal tissue (including those concerning long-term cardiovascular disease or neurotoxicity) were considered to be difficult to perform due to the requirement for long-term follow-up, a rigorous methodology and large patient numbers, in addition to being costly (5–7). The aim of the present study was to quantify this assumption in a systematic review of the literature by identifying the particularly influential scientific publications as well as the areas that are currently predominantly being investigated. For various reasons, including (although not limited to) tenure track or probability of future funding, study groups attempt to publish their results in a way that ensures high visibility and allows for the broad adoption of the progress achieved. The success of a publication may be defined by various factors. The impact factor of journals is a double-edged sword, for example in publication bias exists, where negative or inconclusive studies are not reported (8–10). Article download rates may provide an indication of visibility and impact; however, this depends on the presence and the amount of fees that are charged by the publisher. Another potential measure of the quality and impact of studies is the citation rate (11,12). Notable or practice-changing studies are likely to be cited by follow-up trials, editorials and review articles. The citation rates of articles published between 2006 and 2010 were evaluated for the purpose of the present study. Information regarding highly cited article types may facilitate strategic decision-making and preparation of future research projects. Furthermore, identifying underrepresented research areas may initiate the improvement of resource allocation and increase the focus on these areas.

## Materials and methods

### Data source, search strategy and inclusion criteria

On November 7, 2012, a systematic search of the database, Scopus (Elsevier B.V.; www.scopus.com) using the key words ‘normal tissue’ and ‘radiotherapy’ was performed. The evolution of publication activity following the year 2000 was analyzed in order to provide a broader view of the subject. Articles, including reviews, and clinical and experimental studies, published between 2006 and 2010 were selected regardless of language and article type. Pure dosimetric studies, for example those comparing normal tissue doses with photons versus protons (treatment planning without clinical follow-up data) were excluded.

### Analysis of patterns of citation

Finally, patterns of citation (using the field, ‘times cited’ in the Scopus citation database) were analyzed as described in our previous study (13). The total number of accumulated citations was evaluated (irrespective of their origin) and the proportion of highly cited articles, arbitrarily defined as those with ≥25 citations, was investigated. Due to the notably low number of such articles, the cut-off was lowered to ≥15 citations. A complete list of articles that have been cited ≥15 times may be requested from the corresponding author.

### Statistical analysis

To estimate the longitudinal trends, the estimated annual percentage change was calculated by use of a linear regression model (IBM SPSS Statistics 21, Armonk, NY, USA). Statistical significance was assessed using the two-tailed test. P<0.05 was considered to indicate a statistically significant difference.

## Results

### Review of the literature

**Figure 1:**
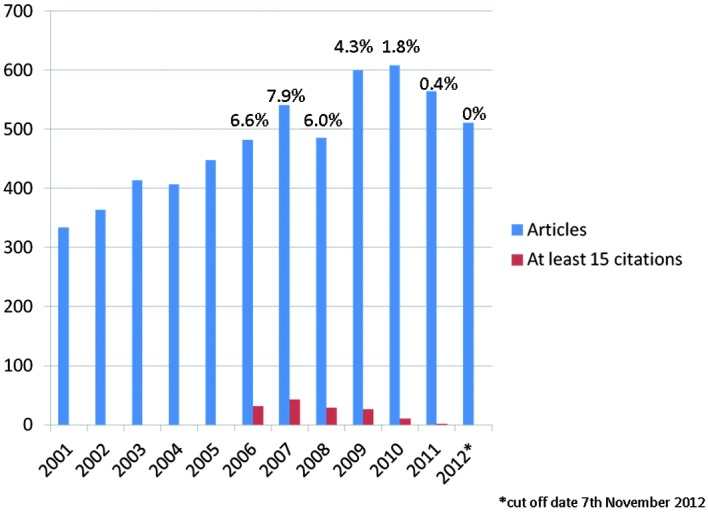
Number of articles published annually. The percentages displayed represent the red bars.

Between 482 and 608 articles per year were published during the five-year period that was investigated. Fig. 1 presents the number of publications per year, which significantly increased between 2006 and 2010 by >50% (P>0.05). In each year <10% of publications accumulated ≥15 citations. Fig. 1 also shows that accumulation of citations takes ≥3–4 years subsequent to publication. Therefore, articles published in 2009 and 2010 were less likely to have accumulated ≥15 citations.

### Most cited references

References (14–38) represent the five most cited articles annually between 2006 and 2010. The most cited articles were published in 12 different scientific journals. Ten articles (40%) were published in the International Journal of Radiation Oncology Biology and Physics, and two (8%) in each of the Journal of Clinical Oncology, Radiotherapy and Oncology, Nature Reviews Cancer and Oncologist. Table I shows the 10 most cited articles overall. The majority of these were reviews or radiobiological modeling studies and all but three were published prior to 2009. Since articles that were published, for example, in 2006 are more likely to have accumulated a large number of citations than articles that were published in 2010, the mean of the annual numbers of citations was also calculated. For this purpose, 2012 was defined as 0.85 years (January 1st-November 7th). Table II displays the 10 articles with the most citations per year and contains articles that were published between 2006 and 2010. The majority of these were also reviews or radiobiological modeling studies.

## Discussion

The aim of the current study was to identify influential and highly cited scientific publications (thereby determining the trends in current research) concerning the pathogenesis, epidemiology, prevention, diagnosis, and treatment of normal tissue toxicity during the five-year period between 2006 and 2010. It was hypothesized that large, clinical toxicity prevention or mitigation studies may complement the technical efforts towards improved dose distribution and organ sparing, and improve quality of life of irradiated cancer survivors. However, large, clinical toxicity prevention or mitigation studies are difficult to conduct due to cost issues and competition for funding, thus, are rare and relatively underrepresented. Following arbitrary decisions regarding which database to search and what keywords to use, a systematic literature search was performed and a broad definition of normal tissue-associated publications was applied (excluding pure treatment planning studies without clinical follow-up data). The citation rate of published articles was subsequently evaluated. Articles that have accumulated a high number of citations are likely to have impressed other clinicians/scientists and, thus, may profoundly influence clinical practice or future developments in the field.

The number of studies performed has increased in the time period that was studied in the present study. In contrast to general radiotherapy publications (39), none of the articles regarding normal tissues achieved >40 citations per year. In our previous study, 15% of all articles accumulated ≥40 citations per year and 42% had between 20 and 39. Most citations per year were recorded for meta-analyses and randomized phase III trials. Notably, the lowest figures were observed for review articles, non-phase III prospective clinical trials and retrospective clinical studies (39). In a recent review of glioblastoma research, the ten articles with the highest number of citations were cited ≥100 times annually (13). Only 1.5% of all glioblastoma articles published between 2006 and 2010 accumulated ≥100 citations, however, ~10% had 25–99 citations. In a previous study regarding radiosurgery for various conditions the same figure was reported (40); this particular study did not include articles with <100 citations. To the best of our knowledge, no other published citation studies have focused on normal tissue research. The results of the present study indicate that considerable differences exist with regards to the topics mentioned above.

In addition to the absolute number of citations, the mean annual citation rate was also evaluated as the exact time course or kinetics of citation are difficult to predict, and vary with the topic and journal (41). The accumulation of citations of recently published articles and the reduced interest in older articles over time presents a challenge if reliable quantitative analysis is to be attempted. The current study did not account for the date of publication in terms of whether an article was published earlier or later during a specific year. For the purpose of this study, the selected methods were considered to be sufficient. However, more detailed and quantitative analyses may be performed with the internet-based tools available. It must be noted that searches using different databases or different key words may result in more or less variable citation counts; therefore, the present results only provide a snapshot. Furthermore, self-citation is likely to influence the final citation count of sparsely cited articles, whereas its impact on highly cited articles may be less pronounced. It was recently estimated that 6.4% of all citations per article (interquartile range, 2.8–11.3; mean, 8.4) were self-citations (42). The studies most vulnerable to this effect were those with a higher number of authors and small sample sizes.

The results of the present study are consistent with the theory that citation rate progressively increases several years after publication. However, the aim of the present study was not to investigate the dynamics of citation counts. As the majority of scientific radiation oncology journals have steadily increased in numbers of published issues and articles, and considering that each article contains a certain number of references, the increase in total publication numbers over time is expected to result in a parallel increase in citation rates. Notably, the highly cited studies were published in a large number of different scientific journals with or without high impact factors and were always in English.

Between 2006 and 2010, significant progress has been achieved in the areas of genomic analyses, toxicity prediction and implementation of highly conformal radiotherapy techniques, which reduce normal tissue doses. Various articles regarding these subjects were among those with the highest numbers of citations (13,26,27,31,32). Systematic reviews were also considered likely to achieve a high number of citations. The large diversity of current research topics covering all clinical, pre-clinical, biological and technical aspects of the field is noteworthy. Prospective clinical research in areas including prevention and mitigation, using radioprotectors and response modifiers, was underrepresented. This is unusual considering the major focus on radiation-induced long-term effects in breast cancer, lymphoma and brain cancer survivors (43–45). Efforts to support awareness, funding and publication of normal tissue studies, in particular clinical strategies that aim to reduce toxicity and improve quality of life, may be warranted.

In conclusion, publication numbers have increased in recent years; however, the number of highly cited articles is limited. In addition to the dose-volume relationship and pathogenesis of normal tissue effects, the predominating research areas were genomic analyses and toxicity prediction. For clinical practice, the development of effective prevention and mitigation strategies is required, as improvements in technology alone cannot prevent all types of radiation-induced toxicity. Radiation fields inevitably include certain amounts of normal tissue, however, current clinical studies primarily focus on cancer cells and efforts to increase their radiosensitivity. Thus, general support and funding for clinical studies focusing on normal tissues are required.

## Figures and Tables

**Table I:** Ten articles with the highest number of citations (absolute count).

Author, year (reference)	Short title	Absolute citation count	Citations per year
Bentzen 2006 (14)	Review of late effects	154	22
François *et al* 2006 (15)	Human mesenchymal stem cell engraftment	123	18
Kong *et al* 2006 (16)	Radiation pneumonitis and fibrosis	112	16
Wazer *et al* 2006 (17)	Late toxicity after breast radiotherapy	90	13
Fiorino *et al* 2009 (29)	Pelvic normal tissue review	67	17
Bentzen and Trotti 2007 (19)	Toxicity of chemoradiation review	66	11
Barnett *et al* 2009 (30)	Tailoring dose by genotype	65	17
Kouvaris *et al* 2007 (20)	Amifostine review	60	10
Kirkpatrick *et al* 2008 (24)	LQ model in radiosurgery	60	12
Michalski *et al* 2010 (34)	Dose-volume effects for rectum	60	21

**Table II:** Articles with the highest number of annual citations.

Author, year (reference)	Short title year count	Citations per	Absolute citation
Bentzen 2006 (114)	Review of late effects	22	154
Michalski *et al* 2010 (34)	Dose-volume effects for rectum	21	60
François *et al* 2006 (15)	Human mesenchymal stem cell engraftment	18	123
Fiorino *et al* 2009 (29)	Pelvic normal tissue review	17	67
Barnett *et al* 2009 (30)	Tailoring dose by genotype	17	65
Kong *et al* 2006 (16)	Radiation pneumonitis and fibrosis	16	112
Bentzen *et al* 2010 (35)	QUANTEC review	15	44
Weiss and Landauer 2009 (31)	Review of radioprotectors	15	59
Zhao and Robbins 2009 (32)	Inflammation and chronic oxidative stress in late normal tissue injury	14	53
Wazer *et al* 2006 (17)	Late toxicity after breast radiotherapy	13	90
